# Pipeline for FlowCam data processing with modular open-source software and optional machine learning classification

**DOI:** 10.7717/peerj.20754

**Published:** 2026-03-24

**Authors:** Katerina Symiakaki, Tim J. W. Walles, Cassidy Park, Gülce Yalçın, Kananat Siangsano, Stella Angela Berger, Jens Christian Nejstgaard

**Affiliations:** 1 Leibniz Institute of Freshwater Ecology and Inland Fisheries (IGB), Stechlin, Germany; 2 Institute of Biology, Freie Universität Berlin, Berlin, Germany; 3 Applied Mathematics, Mahidol University, Bangkok, Thailand

**Keywords:** FlowCam, Plankton, Machine learning classification, Open source software, Imaging, Artificial intelligence, Imaging flowcytometry

## Abstract

Imaging instruments are becoming widely used in plankton research as they offer several advantages over traditional microscopy: (1) processing orders of magnitude more samples and organisms per time, (2) collection of more quantitative trait data from all organisms, (3) reducing human bias including the possibility to reanalyse image data, (4) rapid imaging of samples avoiding bias by deterioration of preserved samples before analysis, and further (5) some imagers allow analysis of live organisms enabling detection and quantification of delicate organisms that cannot be properly fixed. However, processing the huge number of images produced by common plankton imagers such as the FlowCam (Yokogawa Fluid Imaging Technologies, Inc., ME, USA) remains challenging. VisualSpreadsheet (VSP)—the commercial software necessary to operate FlowCam instruments—offers images, associated particle properties, and statistical analysis tools. However, it has licensing costs, runs exclusively on Windows, and offers limited support for older software versions and machine learning classification. Third-party alternatives to VSP for image sorting and classification have shortcomings related to data format and applicability across systems. We developed a freely available, multi-platform modular pipeline for processing FlowCam data from various instruments and VSP versions, while also adding important functionalities. A preprocessing Python script unifies the output of different VSP versions and detects duplicate images. The size range of target particles can be determined by a user-defined threshold, and their individual biovolume is calculated based on a distance map algorithm. The preprocessed data is summarised in a CSV file that can be opened in LabelChecker, the open-source, cross-platform program presented here. LabelChecker displays FlowCam images without transforming the FlowCam’s output format and enables annotation and validation of labels. The processing pipeline can be paired with machine learning approaches for automatic image classification. Classification results are stored in the same CSV file that opens with LabelChecker for easy label validation and further data processing. We demonstrate the workflow of this pipeline with two plankton datasets. We first annotate images and then use them to train a custom shallow, multi-input classification model. Focussing on accessibility, this pipeline (preprocessing, LabelChecker, and machine learning) paves the way for fast and reproducible plankton analysis of FlowCam data, and enables high-throughput analyses adaptable to a wide range of plankton studies. This freely available plankton imaging pipeline can facilitate a wider use of FlowCam instruments and their data, increasing the overall scientific output. Furthermore, its modular design allows for adaptation to data from other plankton imaging systems.

## Introduction

Imaging technology is widely used for plankton and particle analysis alongside classical methods of enumeration and identification, such as manual microscopy, as it offers many advantages. Imaging instruments, such as the FlowCam (Yokogawa Fluid Imaging Technologies, Inc., ME, USA), the PlanktoScope (Pollina et al., 2022), the Imaging FlowCytobot (IFCB; McLane Research Laboratories, Inc., MA, USA), the CytoSense (CytoBuoy b.v., The Netherlands), the Underwater Vision Profiler (UVP) (Picheral et al., 2022), the modular Deep-focus Plankton Imager (mDPI; Greer et al., 2025), or the Dual Scripps Plankton Camera (DSPC; Merz et al., 2021) are valuable quantitative tools for analysing planktonic organisms of various sizes in the lab or *in-situ*. An overview of most available imaging technologies for aquatic systems is given in Dashkova et al. (2017) and Lombard et al. (2019). Many imaging instruments offer the advantage over other methods that they can be used not only on fixed samples but also on live plankton (*e.g*., Jakobsen & Carstensen, 2011), either as collected samples or *in situ*, improving the detection of delicate organisms and structures often missed or misidentified in fixed samples. Additionally, the high sample throughput increases the likelihood of identifying rare organisms (Stanislawczyk, Johansson & MacIsaac, 2018). Moreover, imaging allows reanalysis of images, enabling unbiased data comparison (Culverhouse et al., 2014) and retrospective tracking of specific species, parasitic infections, species interactions, and other previously unknown, overlooked ecological dynamics or traits that are difficult to quantify by classical microscopy (Orenstein et al., 2022). Finally, imaging approaches allow size, shape and other measurements of all individual or colonial plankton organisms or other particles instead of measuring a subset, as is done in classical microscopy. This enables precise calculation of size (Álvarez et al., 2014) and, if coupled with advanced approaches to shape estimation (Moberg & Sosik, 2012), imaging eliminates the reliance on simple geometric form assumptions for biomass calculations (Saccà, 2017).

The large number of images produced by imaging instruments, however, necessitates the use of automated methods to ease the time-consuming classification task. Machine learning techniques, especially convolutional neural networks (CNN), have been developed and applied to plankton image data for the past two decades to improve sorting and classification (Irisson et al., 2022). However, challenges of plankton classifiers such as limited amount of labelled data, high class imbalance, and large data volumes still remain (Eerola et al., 2024).

For supervised learning, a subset of images must be annotated before classification, so a model can be trained. Besides the proprietary software that (usually) comes with imaging instruments, a variety of independent annotation tools (reviewed in Gomes-Pereira et al. (2016)) have been developed to either segment and annotate large images, *e.g*., Seascape (Teixidó et al., 2011), BIIGLE (Langenkämper et al., 2017), MATLAB Image Labeler (The MathWorks, Inc., Natick, MA, USA), or to label images of individual particles such as ZooImage (Grosjean, Denis & Wacquet, 2018), DeepLOKI (Oldenburg et al., 2023), MorphoCluster (Schröder, Kiko & Koch, 2020) and EcoTaxa (Picheral, Colin & Irisson, 2017). Integrated pre-sorting methods to accelerate the initial sorting based on either unsupervised clustering or classification models are available for DeepLOKI, Morphocluster and EcoTaxa. Nevertheless, users still need to provide labelled data to implement pre-sorting approaches of DeepLOKI and MorphoCluster. EcoTaxa is an online platform for labelling image data acquired with many imaging instruments, including the ZooScan, UVP, FlowCam or Planktoscope. The data can be pre-classified by a Random Forest or CNN classifier, and then users (with appropriate assigned roles) can correct or agree with the assigned labels.

Among available imaging instruments, the FlowCam, a flow imaging microscopy device, is most commonly used in aquatic research for lab-based plankton analyses (Sieracki, Sieracki & Yentsch, 1998; Owen et al., 2022). Its applications are primarily focused on microplankton, particularly phytoplankton, and it is one of the most commercially successful instrument for this size range of organisms, alongside the IFCB and the CytoSense. It acquires several images of the flow cell per second—and the particles in it (source images, can be saved if the user wishes) and automatically crops the particles’ regions of interest (images or vignettes), which are saved in ‘collage files’ or individually (in the most recent software version). We refer to the segmented regions of interest as “images” hereafter. Each image acquired by the FlowCam comes with up to 60 measured properties (depending on the software version and user settings) relating to the image itself and the particle in it, a unique feature among imaging instruments. This allows for studying morphology, taxonomy, biomass, and diversity of plankton (Álvarez et al., 2014) and applying functional and trait-based approaches in plankton research (Orenstein et al., 2022). The accompanying software, VisualSpreadsheet® (VSP), is essential for image acquisition and can be used to view, sort, and group (classify) them based on the statistical similarity of their properties. VSP offers semi-automated options for classification based on the statistical similarity of particle properties of each library, which can be useful (Mirasbekov et al., 2021), although not widely used (Owen et al., 2022).

Workflows in VSP can vary, but in principle, users sort the images of each category either into *libraries—*which are used to detect similar particles in the unsorted data—or saved separately in *list files*. However, the fact that *libraries* need to be carefully combined into a single file at the end of processing can lead to errors, and *list files* multiply the datasets. Until recently, the file output made it difficult to use the data in flexible, multiple ways; images were saved in a condensed format within “collage files” and had to be extracted if the users wished to use the data differently, *e.g*., for training classification models (*e.g*., Álvarez, López-Urrutia & Nogueira, 2012). VSP version 6 resolved this limitation by automatically exporting individual images. However, VSP v6 does not support datasets obtained with older VSP versions (v2 or earlier); therefore, data combining for libraries and condensed format of images remains an issue for some users. Another limitation with working with VSP is its licensing restrictions. It mandates that the processing of the data happens at the same location where the samples were processed, which is often not possible in large or international collaborative research projects. Satellite versions of VSP are available, but they come with additional costs, posing barriers for institutions and individuals with limited funds.

To overcome challenges particularly related to FlowCam data, such as data-software compatibility, error-prone and data-duplicative workflows, difficult-to-handle data output, and post-processing reliance on proprietary software, we developed a dedicated processing pipeline. The pipeline includes a preprocessing step for unifying CSV output data from different VSP versions, data cleaning, and biovolume calculation based on Moberg & Sosik (2012), and the LabelChecker, an open-source, cross-platform program for labelling FlowCam-acquired images through a graphical user interface. Optionally, labelled data can be used to train classification models. Data from all VSP versions are supported without any prior transformation (exported either as collage files or separate images) and are handled in a non-destructive way, *i.e*., the raw data remain unaltered. Except for LabelChecker, all components of the pipeline are implemented in Python (v3.12.3). The LabelChecker and the pipeline scripts are available on GitHub at https://github.com/TimWalles/LabelChecker/releases/latest and https://github.com/TimWalles/LabelChecker_Pipeline.

## Materials and Methods

### Pipeline

**Figure 1:**
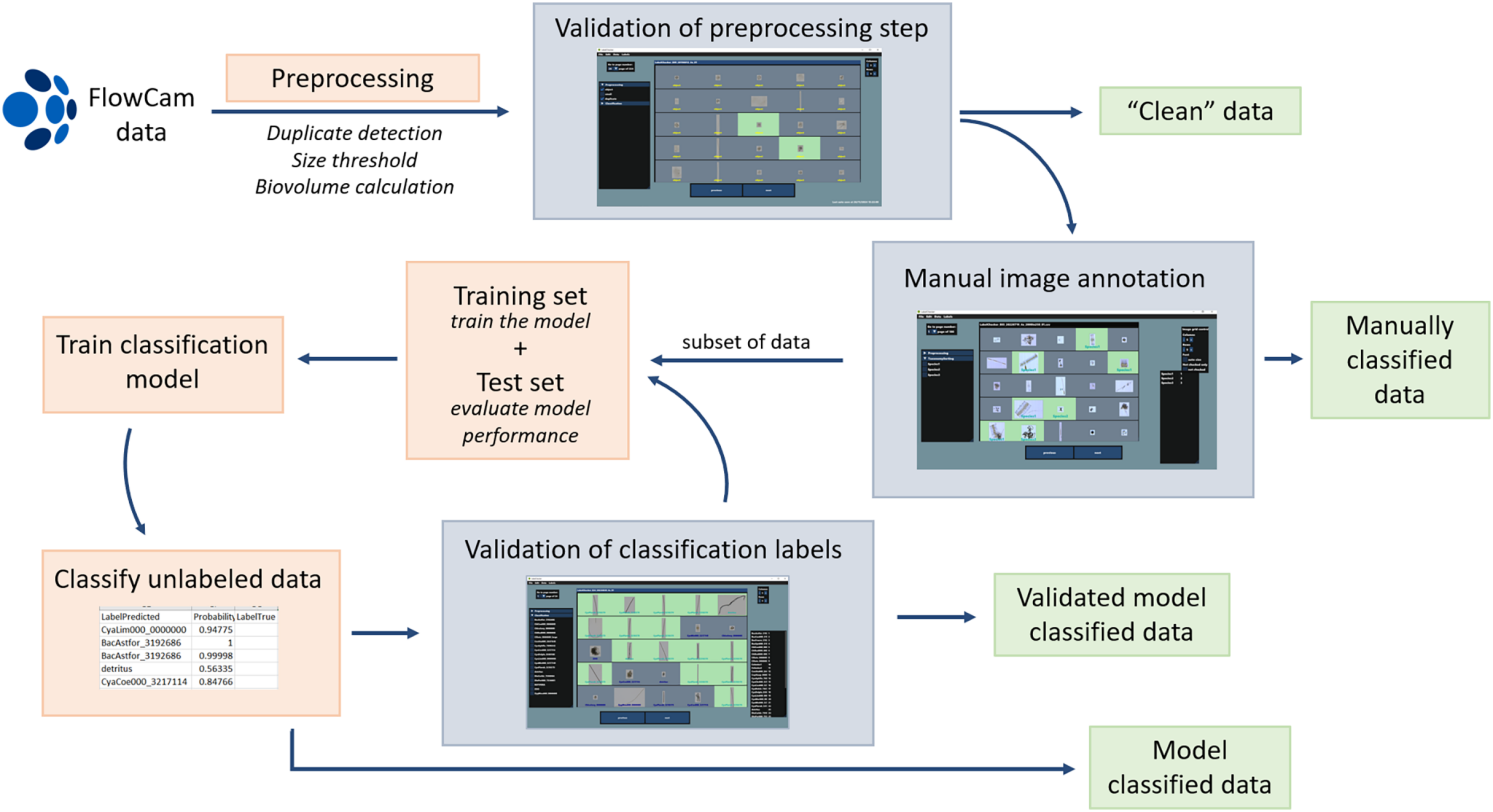
Processing pipeline for FlowCam data. The data processing can end at different steps (clean, duplicate-free data, manually classified data, model-based classified data, validated classified data, see green arrows) depending on user needs and data size and structure. Steps in orange boxes are performed with Python scripts, and steps in grey boxes with the LabelChecker software.

The pipeline consists of two steps, and an optional third step, as described in detail in the sections below (Fig. 1). For clarification, when we refer to classes, we mean the groups the images are assigned to, and not the taxonomic class. (1) FlowCam raw data (images and CSV files of the particle properties) are preprocessed to unify parameter names from different VSP versions, flag duplicate images, and label particles outside a defined size range. Additionally, the script calculates individual particle biovolume based on Moberg & Sosik (2012). The user validates the non-target particles in LabelChecker and can assign further labels if needed. (2) The user initially manually classifies the images within the targeted size range into desired classes in LabelChecker, either of the entire dataset, or optionally (3) of a part of the dataset to create training/test datasets for training a classification model. The model is used to make predictions on unclassified data. In an iterative process, the user validates these predicted data labels and may enrich the training/test dataset. The classification cycle (train model, predict label, correct label, train model, *etc*.) can be repeated until a satisfactory model performance is reached.

For the pipeline to work seamlessly, we recommend saving the FlowCam CSV file with the individual particle properties for each sample automatically during image data acquisition in VSP, ensuring that the file name is the same as the sample run name. We also recommend selecting the option to save binary images automatically (if available, VSP < v6) for a faster and more reliable calculation of the biovolume. Other useful files exported by VSP (not used in our pipeline) are the context CTX file, containing the metadata of sample processing, and the summary CSV or TXT file (depending on VSP version), which includes information such as the imaged volume, necessary for later calculation of particle density in the samples.

### Preprocessing

The data preprocessing involves a Python script that loads, unifies, and saves FlowCam CSV data to ensure compatibility among different FlowCam and VSP versions and the LabelChecker software. The script reads the CSV output of FlowCam data acquired with VSP versions 1.8.92, 2.2.1, 2.4.1, 4.15.1, 4.19.3, 6.0.4 and 6.0.12 and makes the names of measured particle properties uniform.

Sample processing can sometimes produce imaging artefacts such as multiple images of the same particle (hereafter called duplicates) due to sticky particles, clogging, mismatch of image acquisition and particle flow at start or when the pump empties, or poorly set flow rate in comparison to the sample’s density, that usually need to be removed manually (Kydd et al., 2018; Boyette et al., 2018; Owen et al., 2022 and references therein). We differentiate artefacts from non-target particles, since in the latter are successfully imaged particles, but not of interest, such as particles outside the targeted size range or detritus. The preprocessing script includes the distinct data-cleaning components, duplicate detection, and a size threshold. We perform the data-cleaning at an initial stage (rather than during classification) to reduce the number of images for downstream processing. Additionally, an image classifier would struggle to distinguish duplicate images without additional information about the image sequence. The script also includes a trial air bubble detection feature (disabled by default) that can differentiate air bubbles and calibration beads from other imaged particles using a classifier. Users may optionally integrate this functionality into the classification model if desired. All data-cleaning components, as well as the biovolume calculation, can be individually enabled or disabled depending on the user’s choice. An overview of the main preprocessing components is presented on Table S1.

The preprocessing output is a CSV file similar to the FlowCam CSV data output with “LabelChecker_” as a prefix (hereafter LabelChecker_CSV). Rows correspond to images, and columns correspond to particle properties, *i.e*., the parameters provided by the VSP software. The preprocessing step appends seven columns at the end of the file, where automatically-assigned and user-validated labels are stored (from preprocessing and/or classification), as well as the calculated biovolume and surface area.

#### Duplicate detection

This component identifies and flags duplicates by four criteria. (1) The duplicate images appear within five source images from each other. (2) Their source image x-coordinates are less than 100 pixels apart. (3) The bounding boxes of the images have an Intersection over Union of at least 30%, or (4) the set of FlowCam-derived parameters (Edge Gradient, Area based diameter (Abd) Area, Average Blue, Green, and Red intensity, Intensity, Length, Perimeter, Roughness, Sigma Intensity, Transparency, and Width) has a cosine similarity of 99.9% or higher, similar to the approach of Álvarez, López-Urrutia & Nogueira (2012).

#### Size threshold

This preprocessing component flags particles that fall outside the user-defined lower and upper thresholds for size-related parameters measured by the VSP software, such as area-based or equivalent sphere diameter, length, width, *etc*. The size threshold should be set according to the flowcell type and objective combination, pre-screening mesh size, and the size range of the target particles. The label “small” is given to particles below the lower threshold, usually containing too few pixels to allow for reliable identification, and the label “large” to large particles above the upper threshold that are too low in abundance to be quantitatively assessed with the combination of flowcell and sample volume.

#### Biovolume calculation

Besides data-cleaning, the preprocessing script contains a component for biovolume calculation based on Moberg & Sosik (2012). First, the script converts the images to binary (bin) images if they are not exported from the FlowCam or if the images were acquired with VSP < v4 (script adapted from Kraft et al. (2022)). We made this distinction on the version because bin images produced with VSP v4 and higher represent the shapes of organisms more accurately than previous versions. Then, the algorithm uses the binary image to calculate the biovolume according to the Solid of Revolution or Distance Map methods based on two criteria. The Solid of Revolution approach is applied if (1) the ratio between the convex hull area particle and actual area is less than 1.2, or if (2) the eccentricity (calculated from eigenvalues of the covariance matrix of coordinates) is less than 0.8 and the ratio of equivalent circular diameter to major axis length is more than 0.8. These criteria identify roughly circular or moderately elongated organisms that are suitable for the Solid of Revolution method. The transformation of pixels to micrometres is made with the calibration constant provided by the VSP software.

### LabelChecker

**Figure 2:**
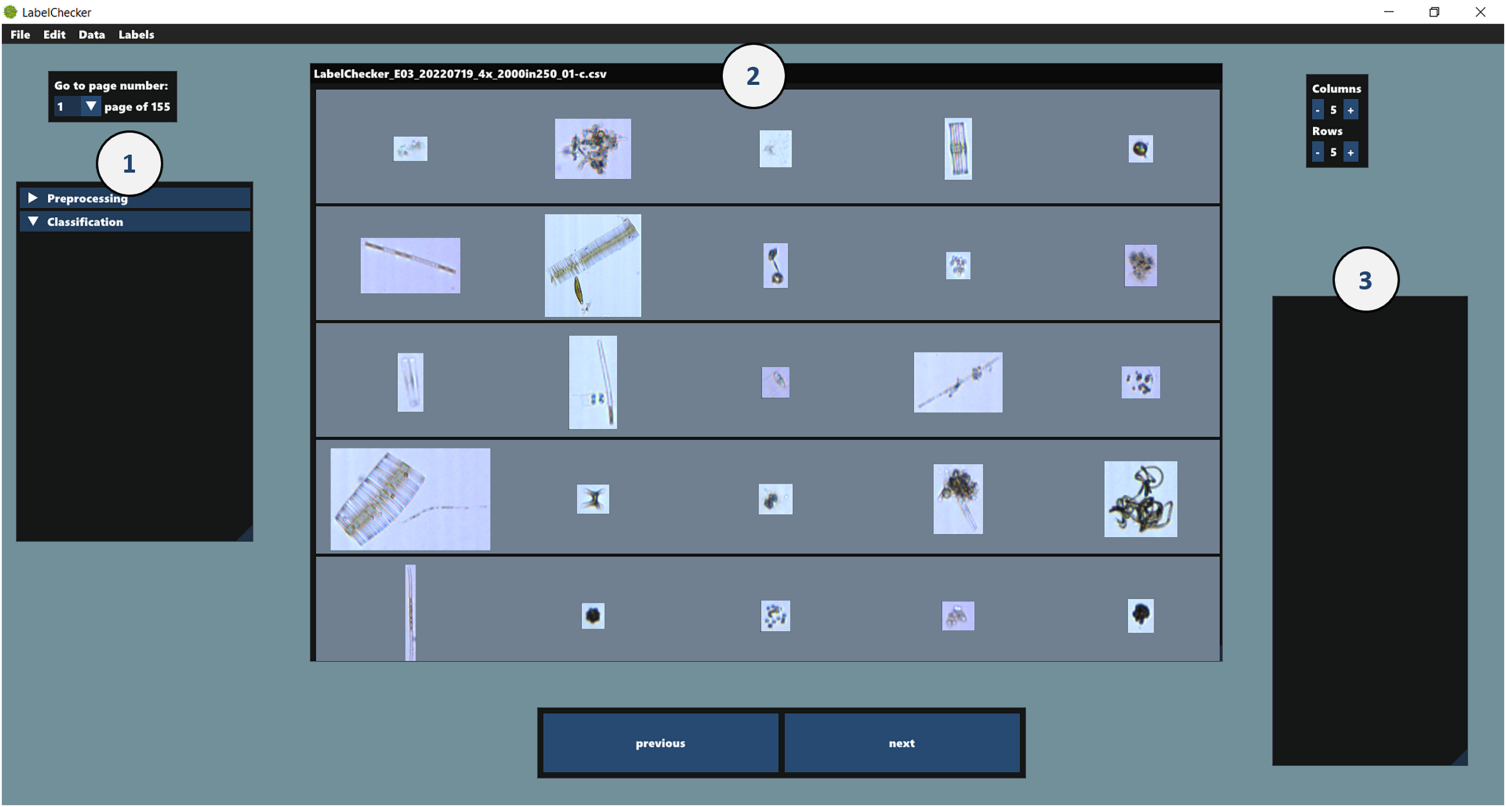
User interface of LabelChecker when a file is open and *Classification* selected. Window 1: Preprocessing or Classification; assigned labels of objects/particles by each step are displayed here. Users can choose to display selected labels with checkboxes. Window 2: Image grid window; images are displayed on a grid and can be selected. Images are highlighted green when the label is validated. Window 3: Stored labels are displayed here with their shortcuts.

LabelChecker is an open-source program developed using the MonoGame framework (MonoGame Foundation, Inc., Dallas, TX) and is compatible with three major operating systems (Windows, MacOS, and Linux). The program works offline, locally, and does not require installation; it can be launched from the corresponding executable file of each operating system. It reads the LabelChecker_CSV file generated by the preprocessing script and presents the individual images on a grid with their original aspect ratios in relation to each other (Fig. 2, window 2).

#### User interface

In LabelChecker, the user can activate the processing tabs *Preprocessing* or *Classification* (Fig. 2, window 1). In the *Preprocessing* tab, users can review and validate the automatic detection of duplicates and particles outside the defined size range, as well as label undetected artefacts. When the *Classification* tab is activated, all images flagged during preprocessing are removed from the display. The user can then either manually label each image or correct the classifier output (see below). A list of assigned labels is stored in a separate CSV file, and individual labels can be selected with a user-defined shortcut (Fig. 2, window 3). All assigned or validated labels are saved in the label-related columns of the LabelChecker_CSV. Regardless of the processing step, users can select and assign or validate labels to multiple images simultaneously. The program autosaves every 5 min to avoid data loss.

#### Features/functionalities

LabelChecker has several features to make annotation easier. Like in VSP, images can be sorted based on their ID, Abd area, diameter, volume, circle fit, length, roughness, transparency, width, probability score (of the classified label—if a model is used) and biovolume. Additionally, the value of each particle property can be displayed on top of each image, as well as information about the file the image comes from. The user can choose to display images of specific classes by clicking on the tick-box next to the class names (Fig. 2, window 1), and between validated or non-validated images only. Lastly, the image grid window (Fig. 2, window 2) can be modified by selecting the number of rows and columns; this affects the number of images displayed and their size (large images get compressed to fit in the grid cell). A magnification feature is also available. An overview of the features and comparison with the most popular alternative software is given in Table S2. The full list of features and functionalities is described in detail in the user manual (https://github.com/TimWalles/LabelChecker/releases/latest).

### Classification model

Users have the option to train and use classification models within the pipeline. To demonstrate the implementation, we constructed a lightweight, multi-input classification model with a focus on quick deployment without specialised computer hardware. The model follows a similar principle as Kerr et al. (2020) by combining a convolutional neural network (CNN) and a multilayer perceptron (MLP) branch that takes images and the properties listed in the LabelChecker_CSV file as input, respectively. Pairs of images and CSV data are fed into the two separate input branches of the model and processed before the branches are concatenated and sent through the final layers for classification. This demo model architecture is presented in Fig. S1.

Before model training, the training datasets were prepared by removing unlabelled objects, non-numeric and date-time columns, columns with the same value for every row, and classes with fewer than five images. Images were resized to 224 by 224 pixels, and batch size was set to 22 images/CSV data pairs. To keep this example model shallow, image augmentation layers were not used. Overfitting was mitigated by dropout regularisation and by only saving models where the validation loss improved. Early stopping and a patience of six epochs were implemented. These parameters can be easily adapted according to the users’ preferences.

### Datasets

To demonstrate the pipeline with LabelChecker and the optional machine learning classification, we used parts of two datasets from mesocosm experiments, acquired with different FlowCam instruments, VSP versions and instrument specifications, and from different systems—one conducted in freshwater and the other in a marine aquatic system.

#### Freshwater plankton dataset of LakeLab experiment

During the summer of 2019, a large-scale enclosure experiment was conducted by exposing the natural plankton community to environmental changes (deep mixing, nutrient addition and different connectivity) at the LakeLab (www.lake-lab.de), an experimental infrastructure permanently installed in Lake Stechlin, Bradenburg, Germany. Integrated water samples from the epilimnion of the LakeLab enclosures were taken by a custom-made hose sampler (Lyche Solheim et al., 2024; Greer et al., 2025), filled into 20 L carboys and transported to shore. Sub-samples of 1 L for FlowCam analysis were immediately taken after gently mixing the carboy. From the subsample, 100 ml of water was gently pre-screened through a 280 µm mesh submerged under water in a beaker. Samples were run live in the FlowCam CYANO equipped with a 4× objective and a 300 µm flow cell in two replicate runs using AutoImage mode. Further specific settings of the FlowCam are listed in Table S3.

#### Marine plankton dataset of SYKE-MRC experiment

During the summer of 2022, an indoor mesocosm experiment was conducted by exposing natural water from the Bay of Finland to warming conditions in the Mesocosm Facility of the Finnish Environment Institute (SYKE), Marine Research Centre (MRC) located in Helsinki, Finland. For phytoplankton enumeration, raw water samples were run live in a FlowCam *vs* with a 10× objective and 100 µm flow cell in two replicate runs using AutoImage mode. Specific settings are listed in Table S3.

## Results

### Freshwater plankton dataset of LakeLab experiment

**Figure 3:**
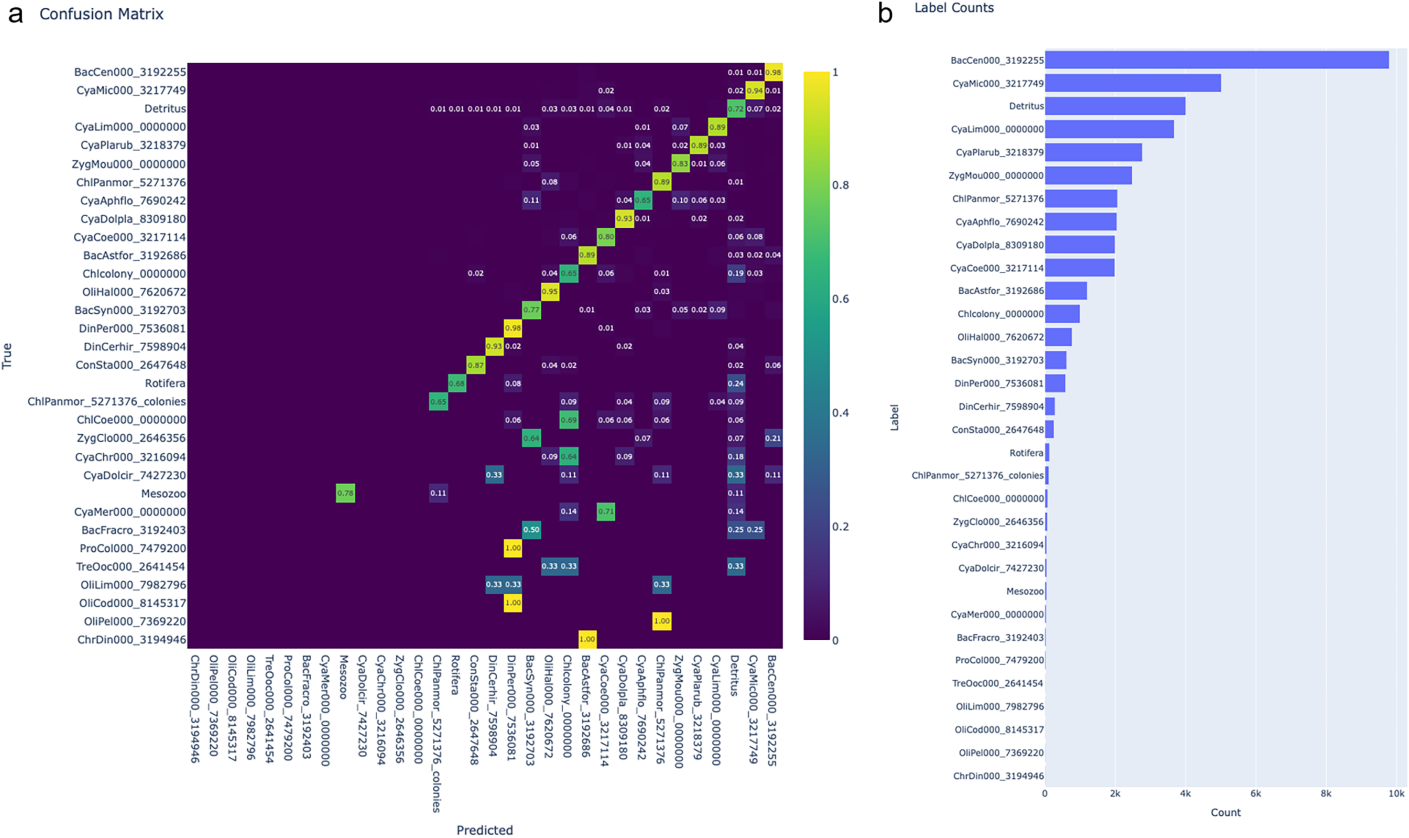
Confusion matrix of the classified test dataset and class distribution of the LakeLab plankton data. (A) Confusion matrix of the classified test dataset. Columns represent the predicted classes, and rows represent the true, validated classes. Colour and the value in each cell indicate the proportion of each predicted class that belongs to the true classes. When the value is 0 the cells are blank (also denoted by the colour). Labels have been sorted by descending number of represented images in the test set. (B) Class distribution of the LakeLab training/test dataset. Counts refer to the number of labelled images per class.

We preprocessed the LakeLab plankton dataset with duplicate detection activated to remove the artefacts, which were validated manually. The size threshold was set on ‘Length’ (particle properties described in the FlowCam and VSP manual), with a 30 µm lower limit and no upper limit (infinity). The targeted objects were manually classified according to Padisák et al. (2023) based on their morphology into 32 classes using LabelChecker, resulting in about 41,202 labelled images for the training-test dataset (the images distribution in the classes is presented in Fig. 3B).

For each class, we assigned a code based on the scientific name and the Global Biodiversity Information Facility (GBIF, https://www.gbif.org) taxon ID. The codes consist of nine letters; the first three of the class the organism belongs to, three for the genus and three for the species (GBIF.org, 2025). The letters are followed by an underscore and the GBIF taxon ID number. When there is no taxon ID on GBIF, the 9-letter code is followed by ‘0000000’. If the label refers only to a genus, the three letters that refer to the species are replaced by ‘000’. *E.g*.,: (class: **Bac**illariophyceae, genus: ***Ast****erionella*, species: ***for****mosa*: BacAstfor_3192686). A full list of the class codes and the corresponding taxa is given in Table S4. Besides the taxon-specific classes, we used general classes for objects that were hard to identify to genus or species level, or fell out of the scope of the analysis, for example: Chlcolony_0000000—colony-forming green algae and some colony-forming cyanobacteria; Rotifera—representatives of the genera *Euchlanis, Kellicottia, Keratella, Polyarthra, Trichocerca*; Mesozoo—crustacean zooplankton and nauplii; Detritus—is here defined as all objects that are not relevant for the specific study, it contains organic material, zooplankton faeces, images too blurry to distinguish, and dead organisms or fragments of organisms.

Training performed in 25 epochs reached 87% accuracy after the training (0.47 loss). The performance of each class is visualised in Fig. 3. The class with the highest F1-score (Table S4) was the diatom *Centronella sp*. M. Voight, 1902 with 98%, followed by the filamentous cyanobacteria *Dolichospermum planktonicum* (Brunnth.) Wacklin, L.Hoffm. & Komárek, 2009, and the colonial cyanobacteria *Microcystis spp*. Lemmermann, 1907, the chlorophyte *Pandorina morum* O. F. Müll, Bory, 1924 and the filamentous cyanobacteria *Planktothrix rubescens* DC. ex Gomont, Anagn. & Komárek, 1988 with over 90%.

### Marine plankton dataset of SYKE-MRC experiment

**Figure 4:**
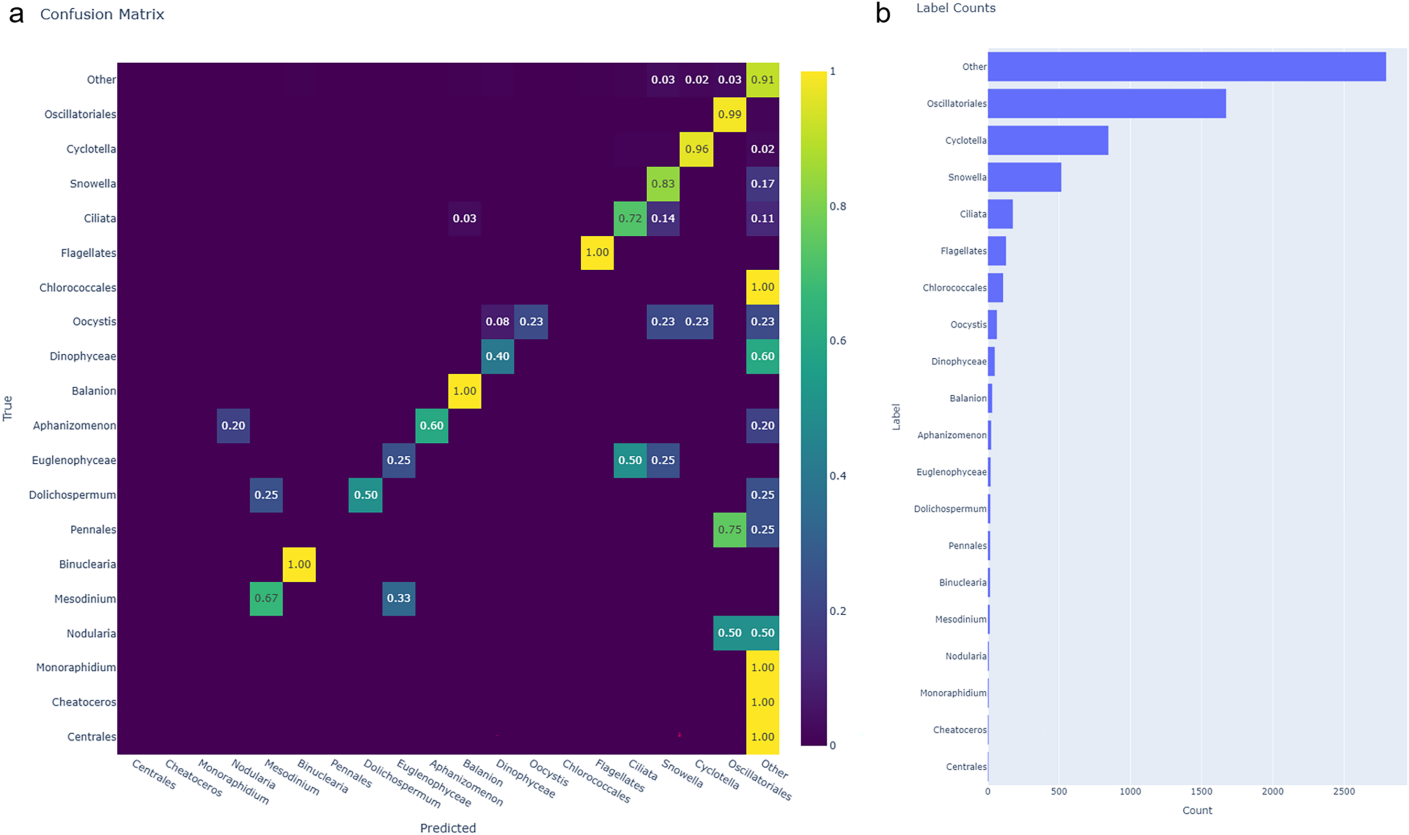
Confusion matrix of the classified test dataset and class distribution of the SYKE-MRC plankton data. (A) Confusion matrix of the classified test dataset. Columns represent the predicted classes, and rows represent the true, validated classes. Colour and the value in each cell indicate the proportion of each predicted class that belongs to the true classes. When the value is 0 the cells are blank (also denoted by the colour). Labels have been sorted by descending number of represented images in the test set. (B) Class distribution of the SYKE-MRC training/test dataset. Counts refer to the number of labelled images per class.

In this case, the preprocessing script was run with different settings to account for differences in the data processing (10× objective compared to 4×, Table S3) and characteristics of the particles in the samples (*e.g*., absence or few filaments). Duplicate detection was deactivated, and the size threshold was set to ‘AbdDiameter’ with a lower limit of 8 μm and no upper limit (infinity). We classified organisms into 20 broader taxonomic groups. We manually sorted 31 files in LabelChecker, which resulted in 6,540 labelled images for the training-test set (distribution presented in Fig. 4B).

The model reached 88% accuracy with the SYKE test set (0.51 loss). We generated a confusion matrix to visualise the performance of each class (Fig. 4). Classes with the highest F1-scores (Table S5), Oscillatoriales, Cyclotella, and Flagellates achieved a near-perfect classification performance. These were also the only classes that were not falsely predicted as ‘Other’. The class Other had a surprisingly high F1-score (89%), but contained predictions that belonged to other classes; in the cases with very low representation in the training-test dataset, Monoraphidium, Cheatoceros, Chlorococcales, and Centrales, 100% of predictions were assigned to Other.

## Discussion

Our proposed pipeline offers accessible, modular components for FlowCam data processing that are adaptable to users’ needs and are compatible with the major computer operating systems. All data is stored in one file per sample, substantially reducing the overall number of files, in contrast to the multiple list or library files produced by VSP.

### Pipeline—preprocessing

In addition to reading and normalising data from all VSP releases, the preprocessing script performs crucial data cleaning services, which usually need to be done manually (*e.g*., Hrycik, Shambaugh & Stockwell, 2019). Each data cleaning service operates independently, allowing users to customise or disable them as needed. The scripts can serve as templates for creating new data-processing components, enabling users to tailor their data preprocessing pipeline to their specific requirements.

The size threshold alone can reduce the proportion of non-target particles and accelerate annotation. Its output depends on the parameters the user defines; for example, setting the threshold on ‘AbdDiameter’ (area-based diameter) performs well on most objects but may misclassify thin, elongated, or filamentous particles as ‘small’ because of the way the parameter is calculated.

The production of artefacts (*e.g*., duplicate images) during sample processing is a common issue, some of which can be avoided with certain methods (Camoying & Yñiguez, 2016; Owen et al., 2022). Nevertheless, it is possible that artefacts are present in the data, and the duplicate detection can be used to easily separate them from target particles. Although this data-cleaning component may currently overperform, meaning there are false positives in the labelled duplicates, we argue that it is easier and faster to correct incorrectly flagged duplicates within a much smaller subset of images than having to locate them within the entire dataset. We therefore recommend carefully considering whether the data-cleaning component should be activated and which settings are necessary according to the specific type of data and analysis selected. Validation of the preprocessing results is necessary, at least until a satisfactory routine has been established for the specific types of samples.

The final preprocessing component is the biovolume and surface area calculation. Biovolume is essential to plankton ecology studies, and it has been frequently addressed (*e.g*., Sieracki, Viles & Webb, 1989; Hillebrand et al., 1999; Borics et al., 2021; reviewed in Saccà (2017)). VSP version 4 and later calculates the biovolume of particles based on the geometric shapes of a cylinder, sphere and prolate spheroid. However, the Moberg & Sosik (2012) method is the most accurate to date for complex shapes. Additionally, we chose to save the surface area that is used in biovolume calculation, since the parameter surface-to-volume ratio is a crucial trait that determines nutrient uptake (Reynolds et al., 2002).

### LabelChecker

A key advantage of using LabelChecker is that it is freely accessible for all users, with no licensing restrictions or reliance on third-party platforms. The software runs locally, allowing users to work on their data anywhere, regardless of network access, or when or where the analysis happened. This flexibility supports collaborative work and promotes the expansion of an active FlowCam user community also in institutions with limited funds, and presently without a FlowCam. Features such as the ability to work on multiple labels at once, several sorting functions, and the use of familiar keyboard shortcuts make the image sorting process fast, intuitive, and user-friendly.

The most popular analytic alternative to VSP, EcoTaxa, also has similar functionalities to LabelChecker, but with some important differences. By operating online, EcoTaxa offers many collaborative features. It allows multiple users to sort images of the same project, so the work can be divided without the need for file sharing. Furthermore, it supports annotation tracking, which allows easy quality control and provides training opportunities (Clayton et al., 2022). However, EcoTaxa requires a TSV file with a specific structure in order to maintain the measurements associated with the particles, which can be time-consuming or complex to create, though there are options to do it automatically (*e.g.*, Jalabert, Lombard & Picheral, 2025 or https://sarigiering.co/posts/from-flowcam-to-ecotaxa/). Additionally, users must upload images of individual particles and define the available particle properties during data import; individual particle images are only exported with FlowCam VSP v6. In contrast, LabelChecker is compatible with FlowCam data output directly, regardless of the VSP version. This simplifies the workflow and reduces the time and effort required for data processing. Lastly, LabelChecker has the potential to be adapted to other plankton imaging platforms, such as the IFCB, by modifying the data reading and loading scripts in the preprocessing. Since particle properties are a unique FlowCam feature, this data could be extracted from images from other instruments (similar to calculations by Álvarez, López-Urrutia & Nogueira (2012)) and populate the LabelChecker_CSV. While this is technically feasible, compatibility depends on the data structure and output format of each instrument, so further development would be needed to ensure the performance is reliable across devices.

### Classification

Our shallow custom model was able to achieve good classification performance despite the challenges of limited images in the training datasets, high class imbalance, and heterogeneous classes (Detritus and Other). The simultaneous production of image and non-image data by the FlowCam permits this dual-input approach, which can be advantageous. Depending on the type of data, the training set of particle properties for the MLP branch can be extended with additional metadata, including *e.g*., time and location of the sample collection (Ellen, Graff & Ohman, 2019). Furthermore, techniques for addressing class underrepresentation, like oversampling and image augmentation (Buda, Maki & Mazurowski, 2018), have the potential to improve the performance of classification models. For simplicity and ease of comparison, our model does not include these techniques, as it is only a demonstration model.

Though we demonstrate the use of machine learning applications in the pipeline with a simple classification model, different, more advanced model architectures can be used, such as the ones highlighted by Bachimanchi et al. (2024). To preserve the functionality of the pipeline across VSP versions, we suggest the model should be compatible with FlowCam’s collage files as input and/or accept CSV data. While individual image files are most commonly used as data input for image classification models by individuals (*e.g*., Álvarez et al., 2014) and platforms, such as the Phytoplankton species classifier by VLIZ (Flanders Marine Institute) or EcoTaxa, this image output format is only recently supported as export by VSP (v6).

EcoTaxa supports training and using classification models with FlowCam data and offers the valuable option of using an existing model to pre-sort imported data. The available models are narrowed down by instrument type, and users need to specify which labels of the chosen model are relevant. However, it is difficult to narrow down the appropriate model with the current naming system, as it does not specify the study system, the type of organism (*e.g*., phytoplankton, zooplankton), or the size distribution of the target organisms. Specifically, datasets and models for freshwater systems are still scarce in the platform (as noted by Pierella Karlusich et al. (2022)), and the outcome of the classification (on phytoplankton) is often not satisfactory (Wang, 2025). Additionally, there is presently limited information about the type and architecture of the model used on the platform or its parameters.

## Conclusion

Our freely available pipeline makes it easier for many FlowCam users to streamline their data processing. Its interoperability and automation enables collaboration and data sharing in a standardised manner between research groups, supporting FAIR-compliant image datasets. The pipeline could potentially be adapted to accommodate data from various laboratory-based or underwater imaging instruments, such as the IFCB, the PlanktoScope, the UVP or the mDPI, facilitating future collaborations among plankton imaging researchers. Such synergistic efforts for image analysis and classification from FlowCam (and potentially other instruments’) users can help accelerate the development of machine learning classification models across systems, leading to better standardisation and comparability between instruments and results, and in turn promoting more FAIR data handling processes in the future. Coupled with pre-sorting tools similar to those used in MorphoCluster (Schröder, Kiko & Koch, 2020), the LabelChecker pipeline could achieve near real-time classification of FlowCam data, as it is already done for the LOKI (Oldenburg et al., 2023) and the IFCB (Kraft et al., 2022).

In conclusion, we propose that the lightweight and adaptable LabelChecker pipeline for FlowCam data, supported with machine learning classification, offers an easily accessible step to promote the necessary development towards reliable high-throughput approaches. Paired with microscopy and molecular techniques, high-throughput imaging supported by machine learning can achieve reliable spatio-temporal qualitative and quantitative analysis of aquatic organisms and other particles. With such approaches broadly adopted in the near future, we will have much stronger tools to understand and forecast the effects of environmental changes and respond with management efforts, including mitigating harmful algae bloom formation, species invasions, and other threats or shifts in aquatic ecosystems.

## Supplemental Information

10.7717/peerj.20754/supp-1 Supplemental Information 1 Architecture of the classification model. Left branch is a Convolutional Neural Network (CNN) with convolutional and rectified linear unit (relu) activation layers. Right branch is a Multilayer Perceptron (MLP) with dense layers with relu activation and dropout layers. The two branches are concatenated before the final dense and dropout layers.

10.7717/peerj.20754/supp-2 Supplemental Information 2 Summary of the services during the preprocessing step of the pipeline: Service, function, description and example.

10.7717/peerj.20754/supp-3 Supplemental Information 3 Comparison of main features of LabelChecker, VSP and EcoTaxa. Note: The comparison is between the display and annotation capabilities of the three software. The table does not include features regarding data acquisition, geospatial data distribution, or taxonomic classification available on these platforms.

10.7717/peerj.20754/supp-4 Supplemental Information 4 Specifications for the instrument and software used to acquire the plankton data.

10.7717/peerj.20754/supp-5 Supplemental Information 5 Classification report on the freshwater phytoplankton LakeLab test set. Taxon: taxon name. Class name: name of the class each image is predicted as. Precision: the proportion of true positives in the total amount of positive model predictions. Recall: the proportion of all positive model predictions that were classified correctly. F1-score: harmonic mean of precision and recall. Num. images: number of images in the test set.

10.7717/peerj.20754/supp-6 Supplemental Information 6 Classification report on the marine phytoplankton SYKE-MRC test set. Class name: name of the class each image is predicted as. Precision: the proportion of true positives in the total amount of positive model predictions. Recall: the proportion of all positive model predictions that were classified correctly. F1-score: harmonic mean of precision and recall. Num. images: number of images in the test set.

## Additional Information and Declarations

### Data Availability

The following information was supplied regarding data availability:

The data to test the pipeline and annotation software are available at Zenodo: Symiakaki, K., Walles, T., Park, C., Yalçın, G., Siangsano, K., Berger, S. A., & Nejstgaard, J. C. (2025). Test data and models for the FlowCam data processing pipeline [Data set]. Zenodo. https://doi.org/10.5281/zenodo.16778650.

The pipeline scripts are available at Zenodo: Walles, T., Symiakaki, K., Siangsano, K., & Park, C. (2025). TimWalles/LabelChecker_Pipeline: LabelChecker processing pipeline v1.0.0 (v1.0.0). Zenodo. https://doi.org/10.5281/zenodo.16785278.

The annotation software is available at Zenodo: Tim Walles, & Symiakaki, K. (2025). TimWalles/LabelChecker: LabelChecker v1.0.6 (v1.0.6). Zenodo. https://doi.org/10.5281/zenodo.16785289.
