# Supplementary figures and images for: Pipeline for FlowCam data processing with modular open-source software and optional machine learning classification
Source: PeerJ. 2026 Mar 24;14:e20754. doi: 10.7717/peerj.20754 (PMC13024276; doi:10.7717/peerj.20754)

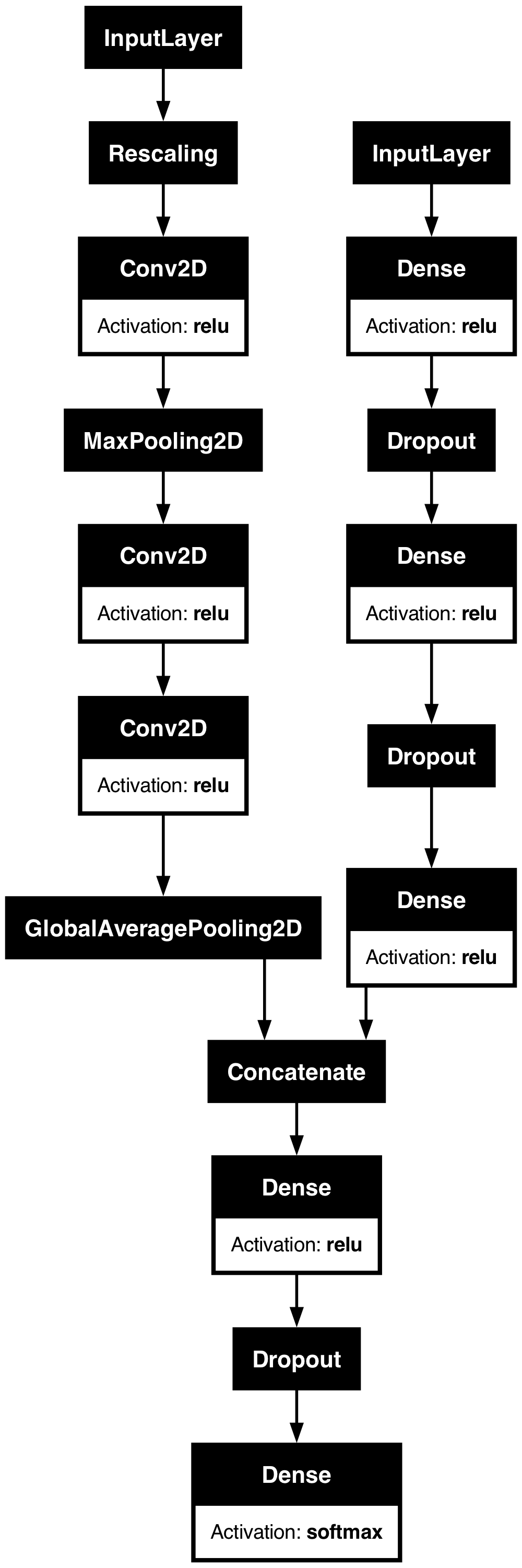

Supplement: Supplemental Information 1 — Left branch is a Convolutional Neural Network (CNN) with convolutional and rectified linear unit (relu) activation layers. Right branch is a Multilayer Perceptron (MLP) with dense layers with relu activation and dropout layers. The two branches are concatenated before the final dense and dropout layers. [file peerj-14-20754-s001.png]
