# Supplementary material for: Pipeline for FlowCam data processing with modular open-source software and optional machine learning classification
Source: PeerJ. 2026 Mar 24;14:e20754. doi: 10.7717/peerj.20754 (PMC13024276; doi:10.7717/peerj.20754)
Supplement: Supplemental Information 2 [file peerj-14-20754-s002.docx]

| **Service** | **Function** | **Description** | **Example** |
| --- | --- | --- | --- |
| **Size threshold** | Filter particles based on their size | Identifies particles that fall outside user-defined lower and upper size limits, labeling them as "small" or "large" based on FlowCam-derived size-related parameters (AbdDiameter, Length, Width, etc.) | If the lower limit is set at AbdDiameter “30” and the upper AbdDiameter “300”, all particles with AbdDiameter < 30 μm will be labeled “small” and those with AbdDiameter > 300 μm “large” |
| **Duplicate detection** | Flag duplicate images of the same particle | Identifies and labels duplicate particle images based on their spatial proximity, bounding box overlap, and similarity in FlowCam-derived parameters. | FlowCam produces duplicates (“several images of the same particles) in case particles get stuck, the pump empties or at the start of a run, these are considered artefacts and can be detected in the preprocessing step |
| **Biovolume calculation** | Calculate individual particle biovolume and surface area | Calculates the biovolume (and surface area) of particles using binary images, with the Solid of Revolution or Distance Map method, depending on shape characteristics. Method developed by Moberg & Sosik, 2012. | This biovolume calculation can be applied to all particles independent of knowing their geometrical shape. This allows a rapid analysis of size class analysis. |
