# Supplementary material for: Pipeline for FlowCam data processing with modular open-source software and optional machine learning classification
Source: PeerJ. 2026 Mar 24;14:e20754. doi: 10.7717/peerj.20754 (PMC13024276; doi:10.7717/peerj.20754)
Supplement: Supplemental Information 3 — Note: The comparison is between the display and annotation capabilities of the three software. The table does not include features regarding data acquisition, geospatial data distribution, or taxonomic classification available on these platforms. [file peerj-14-20754-s003.docx]

| **Feature** | **LabelChecker** | **VisualSpreadsheet (VSP)** | **EcoTaxa** |
| --- | --- | --- | --- |
| **Accessibility** | Free | Licensed | Free |
| **Deployment** | Local | Local (either on FlowCam instrument or satellite version) | Online |
| **VSP version compatibility** | Compatible with all VSP versions between 1.8-6.0 | VSP versions 1 to 4 are readable with Version 4, but only VSP v4-6 is compatible with version 6 | Unknown |
| **Operating system compatibility** | Windows, MacOS and Linux | Windows only | Not applicable (online) |
| **Data Import** | Native output format (any VSP version); requires LabelChecker_CSV generated at preprocessing step | Native output format (VSP version-specific) | Requires individual images; TSV file with specific structure (may have usage limits depending on data size) |
| **Image Sorting** | Multiple labels at once; keyboard shortcuts; filter and sorting functions | Filter and sorting functions; and support by libraries (need to be set-up beforehand, statistical approach) | Multiple labels at once; filter and sorting functions |
| **Particle Property Display** | Yes | Yes | Yes (must be defined during import) |
| **Machine learning classification support** | Yes | No | Yes |
| **Source code** | Open | Not available | Open |
| **User support** | Discord | Via email | Via email |
| **Collaboration** | Moderate (some file sharing required) | Limited (primarily single-user) | Strong (multiple users can collaborate on a project) |
| **Data ownership** | User | User | Unknown |
