# Supplementary material for: Pipeline for FlowCam data processing with modular open-source software and optional machine learning classification
Source: PeerJ. 2026 Mar 24;14:e20754. doi: 10.7717/peerj.20754 (PMC13024276; doi:10.7717/peerj.20754)
Supplement: Supplemental Information 4 [file peerj-14-20754-s004.docx]

|  | **LakeLab** | **SYKE** |
| --- | --- | --- |
| **FlowCam model** | CYANO (8000 series) | VS |
| **Flow-cell** | 300 µm (FOV-300) | 100 µm |
| **Objective** | 4x | 10x |
| **Camera** | SenTech 1.3 Mp Color | SONY_XCD_SC90CR Color |
| **Image acquisition mode** | AutoImage | AutoImage |
| **Frame Rate** | 6.00 fps | 10.00 fps |
| **Pump** | C80 Syringe 5 mL | peristaltic |
| **Flow Rate** | 0.900 mL/min | 0.100 mL/min |
| **Software VisualSpreadsheet** | 4.15.1 | 4.19.3 |
| **Instrument Serial No:** | 10436 | Unknown |
| **Sample run time** | 5 minutes | 10 minutes |
