# Supplementary material for: Pipeline for FlowCam data processing with modular open-source software and optional machine learning classification
Source: PeerJ. 2026 Mar 24;14:e20754. doi: 10.7717/peerj.20754 (PMC13024276; doi:10.7717/peerj.20754)
Supplement: Supplemental Information 5 — Taxon: taxon name. Class name: name of the class each image is predicted as. Precision: the proportion of true positives in the total amount of positive model predictions. Recall: the proportion of all positive model predictions that were classified correctly. F1-score: harmonic mean of precision and recall. Num. images: number of images in the test set. [file peerj-14-20754-s005.docx]

| **Taxon** | **Class name** | **Precision** | **Recall** | **F1-score** | **Num. images** |
| --- | --- | --- | --- | --- | --- |
| Asterionella formosa | BacAstfor_3192686 | 0.87 | 0.89 | 0.88 | 240 |
| Centronella sp. | BacCen000_3192255 | 0.98 | 0.98 | 0.98 | 1958 |
| Fragilaria crotonensis | BacFracro_3192403 | 0.00 | 0.00 | 0 | 4 |
| Synedra spp. | BacSyn000_3192703 | 0.46 | 0.77 | 0.58 | 123 |
| Coelastrum spp. | ChlCoe000_0000000 | 0.00 | 0.00 | 0.00 | 16 |
| Colonies | Chlcolony_0000000 | 0.64 | 0.65 | 0.64 | 200 |
| Pandorina morum | ChlPanmor_5271376 | 0.92 | 0.89 | 0.91 | 412 |
| Pandorina morum colonies | ChlPanmor_5271376_colonies | 0.65 | 0.65 | 0.65 | 23 |
| Dinobryon spp. | ChrDin000_3194946 | 0.00 | 0.00 | 0 | 1 |
| Staurastrum spp. | ConSta000_2647648 | 0.80 | 0.87 | 0.83 | 52 |
| Aphanizomenon flos-aquae | CyaAphflo_7690242 | 0.81 | 0.65 | 0.72 | 408 |
| Chroococcus spp. | CyaChr000_3216094 | 0.00 | 0.00 | 0.00 | 11 |
| Coelosphaerium spp. | CyaCoe000_3217114 | 0.81 | 0.80 | 0.8 | 398 |
| Dolichospermum circinale | CyaDolcir_7427230 | 0.00 | 0.00 | 0 | 9 |
| Dolichospermum planktonicum | CyaDolpla_8309180 | 0.90 | 0.93 | 0.92 | 399 |
| Limnothrix spp. | CyaLim000_0000000 | 0.90 | 0.89 | 0.89 | 735 |
| Merismopedia spp. | CyaMer000_0000000 | 0.00 | 0.00 | 0 | 7 |
| Microcystis spp. | CyaMic000_3217749 | 0.89 | 0.94 | 0.92 | 1004 |
| Planktothrix rubescens | CyaPlarub_3218379 | 0.92 | 0.89 | 0.91 | 553 |
| Ceratium hirundinella | DinCerhir_7598904 | 0.84 | 0.93 | 0.88 | 57 |
| Peridinium sp. | DinPer000_7536081 | 0.82 | 0.98 | 0.89 | 118 |
| Mesozooplankton | Mesozoo | 0.88 | 0.78 | 0.82 | 9 |
| Codonella sp. | OliCod000_8145317 | 0.00 | 0.00 | 0 | 3 |
| Halteria spp. | OliHal000_7620672 | 0.67 | 0.95 | 0.79 | 155 |
| Limnostrombidium spp. | OliLim000_7982796 | 0.00 | 0.00 | 0 | 3 |
| Pelagotrombidium spp. | OliPel000_7369220 | 0.00 | 0.00 | 0 | 1 |
| Coleps sp. | ProCol000_7479200 | 0.00 | 0.00 | 0 | 4 |
| Rotifera | Rotifera | 0.81 | 0.68 | 0.74 | 25 |
| Oocystis spp. | TreOoc000_2641454 | 0.00 | 0.00 | 0 | 3 |
| Closterium spp. | ZygClo000_2646356 | 0.00 | 0.00 | 0 | 14 |
| Mougeotia spp. | ZygMou000_0000000 | 0.79 | 0.83 | 0.81 | 496 |
| Detritus | Detritus | 0.80 | 0.72 | 0.76 | 800 |
|  |  |  |  |  |  |
|  | accuracy |  |  | 0.87 | 8241 |
|  | macro avg | 0.50 | 0.52 | 0.51 | 8241 |
|  | weighted avg | 0.86 | 0.87 | 0.86 | 8241 |
