# Supplementary material for: Pipeline for FlowCam data processing with modular open-source software and optional machine learning classification
Source: PeerJ. 2026 Mar 24;14:e20754. doi: 10.7717/peerj.20754 (PMC13024276; doi:10.7717/peerj.20754)
Supplement: Supplemental Information 6 — Class name: name of the class each image is predicted as. Precision: the proportion of true positives in the total amount of positive model predictions. Recall: the proportion of all positive model predictions that were classified correctly. F1-score: harmonic mean of precision and recall. Num. images: number of images in the test set. [file peerj-14-20754-s006.docx]

| **Class name** | **Precision** | **Recall** | **F1-score** | **Num. images** |
| --- | --- | --- | --- | --- |
| Balanion | 1 | 0.29 | 0.44 | 14 |
| Binuclearia | 0.5 | 0.2 | 0.29 | 5 |
| Chlorococcales | 1 | 0.05 | 0.1 | 19 |
| Ciliata | 0.46 | 0.8 | 0.59 | 15 |
| Cyclotella | 0.92 | 0.99 | 0.96 | 144 |
| Dinophyceae | 0.71 | 0.29 | 0.42 | 17 |
| Euglenophyceae | 0.5 | 0.18 | 0.27 | 11 |
| Flagellates | 0.95 | 0.95 | 0.95 | 20 |
| Oocystis | 0.92 | 0.63 | 0.75 | 19 |
| Oscillatoriales | 0.98 | 1 | 0.99 | 1112 |
| Other | 0.84 | 0.94 | 0.89 | 315 |
| Pennales | 0 | 0 | 0 | 20 |
| Snowella | 0.91 | 0.87 | 0.89 | 130 |
|  |  |  |  |  |
| accuracy |  |  | 0.93 | 1841 |
| macro avg | 0.75 | 0.55 | 0.58 | 1841 |
| weighted avg | 0.93 | 0.93 | 0.92 | 1841 |
